# Altered Blood Molecular Markers of Cardiovascular Function in Rats after Intrauterine Hypoxia and Drug Therapy

**DOI:** 10.3390/cimb45110547

**Published:** 2023-10-30

**Authors:** Olena Popazova, Igor Belenichev, Oleh Yadlovskyi, Valentyn Oksenych, Aleksandr Kamyshnyi

**Affiliations:** 1 Department of Histology, Cytology and Embryology, Zaporizhzhia State Medical and Pharmaceutical University, 69000 Zaporizhzhia, Ukraine; 2 Department of Pharmacology and Medical Formulation with Course of Normal Physiology, Zaporizhzhia State Medical and Pharmaceutical University, 69000 Zaporizhzhia, Ukraine; 3 Institute of Pharmacology and Toxicology, National Medical Academy of Ukraine, 03057 Kyiv, Ukraine; 4 Broegelmann Research Laboratory, Department of Clinical Science, University of Bergen, 5020 Bergen, Norway; 5 Department of Microbiology, Virology and Immunology, I. Horbachevsky Ternopil State Medical University, 46001 Ternopil, Ukraine

**Keywords:** prenatal hypoxia, cardioprotective, heat shock proteins, Angiolin, L-arginine, Thiotriazoline, Mildronate, HSP70, HIF-1, eNOS

## Abstract

Many children and adults who have suffered prenatal hypoxia at an early age develop many serious diseases. This disease is an actual problem of pediatric cardiology and little studied. The aim was to analyze the cardioprotective effect of L-arginine, Thiotriazoline, Angioline, and Mildronate on the cardiovascular system of rats after prenatal hypoxia. Methods: The experiments were carried out on 50 female white rats; intraperitoneal sodium nitrite solution was administered daily to pregnant female rats after 16 days at a dose of 50 mg/kg. Control pregnant rats received saline. The offspring were divided into groups: 1—intact; 2—the control group of rat pups after PH, treated daily with physiological saline; 3—six groups of rat pups after PH, treated daily from the 1st to the 30th day after birth. Heat shock protein HSP70 was determined by enzyme immunoassay, ST2 Nitrotyrosine, and eNOS was observed by ELISA. Results: Angiolin showed a high cardioprotective effect even a month after discontinuation of the drug, and after introduction, the highest decrease in ST2 nitrotyrosine was revealed. Thiotriazoline and L-arginine have an antioxidant effect and a positive effect on eNOS expression, increasing the concentration of HSP70. Mildronate increased the expression of eNOS and the concentration of HSP70 in the blood of experimental rats after a course of administration, but did not show an antioxidant effect and did not reduce the concentration of nitrotyrosine. The results obtained indicate the cardioprotective effect of modulators of the NO system with different mechanisms of action of drugs after prenatal hypoxia.

## 1. Introduction

Post-hypoxic disorders of the cardiovascular system occupy one of the leading places in the structure of morbidity in newborns, occurring, according to various sources, in 40–70% infants who have experienced prenatal hypoxia. These disorders contribute significantly to a multitude of often severe ailments in both children and adults [1,2,3]. The mechanisms underlying the emergence of post-hypoxic cardiac disorders have received limited investigation, rendering them a pressing concern within pediatric cardiology. The clinical symptoms of this pathology in the acute period are polymorphic, often disguised as other diseases, and it is often necessary to carry out differential diagnostics with congenital heart defects, congenital carditis, and cardiomyopathies [4,5].

The above findings contribute to the identification of novel structural, molecular, and biochemical characteristics associated with post-hypoxic cardiovascular disorders in newborns. In light of these discoveries, the development of innovative approaches to drug therapy emerges as a crucial objective within contemporary pharmacology.

In current perspectives, endothelial dysfunction and related NO system disturbances form the basis for the onset of numerous cardiovascular conditions [6,7]. Nonetheless, scant published data exist elucidating the role of the NO system in the onset of cardiovascular pathologies in newborns and the potential cardioprotective effects offered by its modulators. Several studies have established the cardio- and endothelioprotective properties of drugs that can both increase the synthesis of NO and the bioavailability of this messenger [8].

L-arginine, Thiotriazolin, Angiolin, and Mildronate captured our attention as potential cardioprotective agents with NO-mimetic effects. L-arginine acts as a substrate for NO generation within vascular endothelial cells and showcases antioxidant, cytoprotective, antihypoxic, and membrane-stabilizing characteristics [9,10,11]. Thiotriazoline (morpholinium 3-methyl-1,2,4-triazolyl-5-thioacetate) acts as a selective scavenger for NO and its cytotoxic derivatives, elevating NO bioavailability. Thiotriazoline demonstrates cardioprotective, anti-ischemic, and antioxidative characteristics [12,13,14,15]. 

Angiolin ([S]-2,6-diaminohexanoic acid 3-methyl-1,2,4-triazolyl-5-thioacetic acid) enhances eNOS expression, elevates NO bioavailability, increases VEGF expression, and augments the density of proliferating endothelial cells in muscle-type vessels and microcirculatory channels. It showcases endothelio-, cardio-, and neuroprotective characteristics, along with antioxidative and anti-ischemic properties [16]. 

Mildronate also impacts NO synthesis through the elevation of the carnitine precursor, gamma-butyrobetaine. It diminishes carnitine-mediated transport of long-chain fatty acids across mitochondrial membranes while leaving the metabolism of short-chain fatty acids unaffected. Additionally, it activates an alternative energy production system—glucose oxidation [17,18]. Mildronate exhibits anti-ischemic and cardioprotective properties [19,20]. 

The aim of the research is to perform an initial evaluation of the cardioprotective impact of L-arginine, Thiotriazoline, Angiolin, and Mildronate on molecular markers within the cardiovascular system of rats following prenatal hypoxia.

## 2. Materials and Methods

### 2.1. PH Experimental Model and Laboratory Animal Characteristics

Zaporizhzhia State Medical University Commission on Bioethics approved the experimental study (protocol No. 33 of 26 June 2021).

Fifty outbred white rat females and ten males, each weighing 220–240 g and aged 4.5 months, were used in the studies. They were taken from the vivarium of the Institute of Pharmacology and Toxicology of the National Medical Academy of Ukraine. The rats lived in typical vivarium settings, which included 20–25 °C, 50–55% humidity, daylight, a feed that was appropriate for this species of laboratory animals, and unlimited access to water. The chronic hematic nitrite-induced PH model was employed in this study. This model results in histological, morphometric, and metabolic irregularities in the offspring’s heart [21,22]. For a fixed term of pregnancy, mature male rats were placed with virgin female rats with a ratio of 2 males per 4 females. The pregnancy period was counted starting from the discovery of spermatozoids in the vaginal smear (day 1 of the pregnancy). Modelling hematic hypoxia was performed in the prenatal period of development by daily intraperitoneal administration of sodium nitrite solution to pregnant female rats from day 16 to day 21 of the pregnancy at 50 mg/kg (the dose causing moderate hypoxia) [23]. Control pregnant rats received physiological solution in the same regime. The progeny was divided into groups: 1, healthy pups from females with physiologically normal pregnancy which received physiological solution; 2, control group of pups after PH which received physiological solution daily; 3, 6 groups of pups after PH that received drugs daily from postnatal day 1 to day 30. The doses of Arginine and Mildronate were found in the public domain, while the doses of Thiotriazoline and Angiolin were calculated experimentally in preclinical trials.

### 2.2. Justification for the Selected Drugs and Their Characteristics

We selected medicines that have been shown in experiments to affect the NO system:

1. Intact group (rats born from rats with uncomplicated pregnancies), physiological solution.
2. Control group (rats born after experiencing intrauterine hypoxia), physiological solution.
3. Thiotriazoline (Morpholinium-3-methyl-1,2,4- triazolyl-5-thioacetic acid) (2.5% injection solution, “Arterium”, Ukraine), metabolitotropic cardioprotector and antioxidant, administered at 50 mg/kg via intraperitoneal injection [24].
4. Angiolin ([S]-2,6-diaminohexane acid 3-methyl-1,2,4-triazolyl-5-thioacecate) (substance, RPA “Farmatron”, Ukraine), anti-ischemic, endothelium-protective drug, administered at 50 mg/kg via intraperitoneal injection [25].
5. L-arginine (42% injection solution in vial, Tivortin, Yuria-pharm, Ukraine), a NO precursor; it mitigates disruptions in the nitroxidergic system in ischemia, administered at 200 mg/kg via intraperitoneal injection [26].
6. Mildronate (2-(2-carboxyethyl)-1,1,1-trimethylhydrazinium) (10% injection solution in ampoules, Grindex (Latvia)), metabolitotropic agent, administered at 100 mg/kg via intraperitoneal injection [27].

Rats were euthanized on days 30 and 60 of the experiment under thiopental anesthesia (40 mg/kg). Blood samples were collected from the celiac artery for further studies.

### 2.3. Preparation of Biological Material

Blood was taken from the abdominal aorta by syringe, and serum was separated by centrifugation at +4 °C at 1500 rpm for 20 min [28] on an Ependorff 5804R centrifuge. The apical part of the heart was placed in Bouin’s fixative for 24 h. After a standard procedure of tissue dehydration and its impregnation with chloroform [29] and paraffin, the myocardium was cast in paraplast (MkCormick, Hunt Valley, MD, USA). Serial histological sections 5 μm thick were prepared on a Microm-325 rotational microscope (Microm Corp., Munich, Germany), which were then used for real-time PCR studies after treatment with o-xylene and ethanol.

### 2.4. Enzyme-Linked Immunoassay

The technique is based on solid-phase enzyme-linked sandwich immunosorbent assay. The level of the heat shock protein HSP70 was measured in the blood serum by enzyme-linked immunoassay using AMP’DR HSP70 high-sensitivity ELISA kit # ENZ-KIT-101-0001, Enzo (Solna, Sweden). The concentration of HSP70 was expressed in ng/mL. Also, the molecular marker of myocardial damage ST2 protein was determined in the blood serum by the solid-phase immunosorbent sandwich ELISA method using the Critical Diagnostics Presage^®^ ST2 Assay kit (REF# BC-1065, San Diego, CA, USA). The concentration of ST2 was expressed in ng/mL. The activity of endothelial NO synthase (eNOS) was determined in blood serum by enzyme immunoassay (Cloud-Clone Corporation kit, #PAA868Ra01, Katy, TX, USA). The concentration of eNOS was expressed in pg/mL. Nitrotyrosine was determined by sol-id-phase immunosorbent sandwich ELISA, using an ELISA Kit (Cat. No. HK 501-02, Uden, The Netherlands) from Hycult Biotech, and expressed in nM/mL.

### 2.5. Polymerase Chain Reaction in Real-Time

Real-time PCR (Polymerase Chain Reaction) amplification was performed to quantify the expression levels of HIF-1 mRNA, using Maxima SYBR Green/ROX qPCR Master Mix (2x) (ThermoScientific, Waltham, MA, USA) with gene-specific primers on a Biorad CFX 96 Real-Time PCR Detection System. The reaction mixture contained 10 µL of 2x Maxima SYBR Green/ROX qPCR Master Mix, 0.5 µL of each gene-specific primer, 2 µL of cDNA template, and nuclease-free water to a final volume of 20 µL. The PCR cycling conditions involved initial denaturation at 95 °C for 10 min, followed by 45 cycles of denaturation at 95 °C for 15 s, primer annealing at 60 °C for 40 s, and elongation at 72 °C for 40 s. The registration of fluorescence intensity occurred automatically at the end of the elongation stage of each cycle through the automatic SybrGreen channel.

The actin beta (*Actb*) gene was used as the reference gene to normalize the expression levels of the target genes. The expression levels of the target genes were quantified relative to the expression of the housekeeping gene using the comparative Ct (2^−ΔΔCt^) method. The Ct values were converted to relative expression values using the formula 2^−ΔCt^, where ΔCt = (Ct target gene − Ct housekeeping gene). The relative expression values were then converted to Log2 values using the formula Log2 (relative expression).

### 2.6. Statistical Analysis

Experimental data were statistically analyzed using “StatisticaR for Windows 6.0” (StatSoft Inc., Tulsa, OK, USA, № AXXR712D833214FAN5), “SPSS16.0”, and “Microsoft Office Excel 2010” software. Prior to statistical tests, we checked the results for normality (Shapiro–Wilk and Kolmogorov–Smirnov tests). In the normal distribution, intergroup differences were considered statistically significant based on the parametric Student’s *t*-test. If the distribution was not normal, the comparative analysis was conducted using the non-parametric Mann–Whitney U-test. To compare independent variables in more than two selections, we applied ANOVA dispersion analysis for the normal distribution and the Kruskal–Wallis test for the non-normal distribution. To analyze correlations between parameters, we used correlation analysis based on the Pearson or Spearman correlation coefficient. For all types of analysis, the differences were considered statistically significant at *p* < 0.05 (95%).

## 3. Results

As a result of the research, it was found that in rats that underwent intrauterine hypoxia, 1 month (6.28 times) after birth and 2 months (3.63 times) after birth, a significant increase in the specific cardiomarker ST2 was observed in the blood. ST2 (Suppression of tumorogenicity 2, Growth Stimulation expressed gene 2, stimulating growth factor expressed by gene 2, also known as IL1RL1) is a member of the IL-1 receptor superfamily. ST2 is the IL-33 receptor. ST2 is a marker of fibrosis and remodeling of cardiac tissue, released by cardiomyocytes and fibroblasts [30]. An increase in ST2 concentration may indicate the formation of ischemic cardiomyopathy, myocardial remodeling, and impaired contractile function of the heart. As can be seen from Table 1 and Table 2, even in the second month of life, the concentration of this marker remains quite high.

Our investigations revealed a persistent elevation in nitrotyrosine levels within the blood of rats following intrauterine hypoxia exposure. Specifically, in 1-month-old rats, the increase was approximately 5.5 times, while in 2-month-old rats, it reached 3.8 times (as detailed in Table 1 and Table 2). We also found a persistent decrease in eNOS expression after intrauterine hypoxia—2.5 times in 1-month-old animals, and 2-fold in 2-month-old animals.

Our research unveiled a consistent decrease in the expression of the 70 kDa heat shock protein (HSP_70_) post-intrauterine hypoxia. This decrease was approximately 5.6 times in 1-month-old animals and 3.44 times in 2-month-old animals. We concluded that intrauterine hypoxia causes intrauterine programming of the hsp70 gene, which leads to inhibition of its response to warm stress and loss of endogenous cardioprotection at a later age. As a result of the study, we found suppression of HIF-1 mRNA expression in the heart in 1-month-old animals by 79% and in 2-month-old animals by 61.1%. 

Thus, the administration of drugs to animals after prenatal hypoxia for 30 days had a therapeutic effect in varying degrees of severity, both immediately and a month after the end of their administration. Angiolin showed the most significant therapeutic effect. Thus, in groups of animals, when it was used, a decrease in ST2 by 77% was found immediately after the end of its administration compared with the control, and a month after the cessation of the administration of Angiolin, the ST2 values in this group did not differ statistically from the values of the intact group. All this indicates a significant cardioprotective effect of Angiolin. The introduction of Angiolin led to a significant increase in eNOS expression by 3.74 times immediately and 2.5 times a month after withdrawal compared with control and by 58.8% and 153.8% compared with intact during a decrease in the level of nitrotyrosine (a decrease by 56.5% immediately and the normalization of this indicator a month after administration compared with the control). 

Angiolin increased the expression of HSP70 (an increase of 2 times immediately and an increase of 1.84 times a month after administration in relation to the control). Angiolin increases HIF-1 mRNA expression 23-fold in the heart of 1-month-old animals subjected to intrauterine hypoxia and 8.2-fold in 2-month-old animals. A positive effect on the NO system and a decrease in oxidative stress during an increase in mRNA HIF-1 and HSP70 seem to provide Angiolin with its cardioprotective effect after intrauterine hypoxia. It should be noted that the cardioprotective effect of Angiolin persisted even one month after discontinuation of the drug. Similar in direction, but less pronounced effect was observed with the introduction of Thiotriazoline and L-arginine. Thus, in the groups receiving Thiotriazoline, there was a significant decrease in ST2 by 68.2% and 66.2% in accordance with the observation period, and in the groups receiving L-arginine, this indicator decreased by 73% and 55.3%, respectively.

The introduction of Thiotriazoline and L-arginine had an antioxidant effect and led to a decrease in oxidative stress. Thus, in the groups receiving Thiotriazoline, there was a significant decrease in nitrotyrosine by 41% and 48% in accordance with the terms of observation, and in the groups receiving L-arginine, this indicator significantly decreased by 22% only immediately after a 30-day administration of the drug. Thiotriazoline and L-arginine had a positive effect on eNOS expression. Thus, in the group treated with L-arginine, the eNOS index returned to normal immediately after discontinuation of the drug, and its expression increased by 65.5% compared with the control one month after discontinuation of the drug. The introduction of Thiotriazoline provided a significant increase in eNOS expression by 75.3% and 38% in accordance with the observation period. The administration of Thiotriazoline and L-arginine significantly increased the concentration of HSP_70_ in the blood of rats after intrauterine hypoxia for different periods of observation—L-arginine immediately after the cessation of its administration (by 124% compared with the control), and Thiotriazoline 30 days after the cessation of its administration (by 53% compared to control). Thiotriazolin significantly increased HIF-1 mRNA expression both in 1-month-old animals and most significantly (6.2-fold compared to control) in 2-month-old animals. L-arginine administration significantly increased HIF-1 mRNA expression immediately after drug withdrawal (9-fold compared with control). Apparently, this is due to the effect on different parts of the NO/SH-mechanism of triggering HIF-1 mRNA expression.

Mildronate had a significant cardioprotective effect during the course of its administration to rats after intrauterine hypoxia—a decrease in ST2 by 69.5% and 50.6% for various periods of observation. According to the degree of influence on this indicator, Mildronate did not differ from L-arginine and Thiotriazoline, but was inferior to Angiolin. Mildronate showed no antioxidant effect and did not reduce the concentration of nitrotyrosine in the blood of rats after intrauterine hypoxia. Mildronate increased the expression of eNOS in the blood of experimental rats only immediately after the course of administration, and a month after the drug was discontinued, the effect was not significant. Mildronate also significantly increased the concentration of HSP70 and the expression of HIF-1 mRNA within a month after the end of the course. According to the degree of influence on this indicator, Mildronate was inferior to L-arginine and Angiolin, remaining a competitor to Thiotriazoline. 

## 4. Discussion

PH modeling leads to the development of postnatal heart disease. In our previous study, it was found that the use of this model of PH leads to a decrease in myocardial contractility and sinus node dysfunction. Cells with signs of apoptosis and dystrophy are detected in the contractile myocardium and conduction system with a certain correlation between the severity of morphological changes and bioelectric rhythm and conduction disorders [31]. The basis of pathological processes in the action of sodium nitrite is hypoxia of mixed genesis, hemic hypoxia due to the formation of methemoglobin, which is combined with tissue hypoxia due to dissociation of oxidation and phosphorylation processes. Disturbance of blood oxygen transport function in pregnant female rats leads to impaired uteroplacental circulation [23,32] and, as a consequence, oxygen starvation of the fetus or embryo. Administration of sodium nitrite at a dose of 50 mg/kg leads to hypoxia of medium severity in adults, according to the criteria proposed by N.F. Ivanitskaya [33]. Also, a number of works have obtained results indicating that exposure to sodium nitrite in the prenatal period can lead to posthypoxic heart damage, ECG [31] myocardial ultrastructure (increase in the proportion of irreversibly damaged mitochondria in the CMC of the intramural zone of the right ventricle) [34], and contractile changes [35,36]. In both newborn and adult animals, our model allows for the evaluation of physiological development in offspring and the effectiveness of experimental cardioprotective therapy after prenatal hypoxia (PH). Administration of sodium nitrite to pregnant rats results in increased methemoglobin levels [37], and specifically hypoxic damage to fetal target organs.

The end result of hypoxic heart damage can be focal dystrophy [38]. We have confirmed this in that study. This was confirmed by molecular methods, namely, the increase in ST2 concentration in the blood of animals after PH. ST2 is a highly sensitive marker of myocardial remodeling, and the risk of heart failure development can be increased in prenatal hypoxia (pre-eclampsia) [39].

Our data on the increase in nitrotyrosine levels after PH do not contradict the results of other researchers, who found an increase in cardiac oxidative stress after intrauterine hypoxia in both male and female rats exposed to intrauterine chemicals [40]. It is also known that increased oxidative stress is closely associated with cardiovascular diseases such as hypertension and coronary heart disease and causes hypertrophy, fibrosis, and apoptosis, leading to impaired cardiac function [41]. 

Oxidative stress in the antenatal period may be a consequence of damage to mitochondria during hypoxia, which makes them a source of ROS [1]. The data obtained by a number of researchers demonstrate that in rats after intrauterine hypoxia, there is an increase in the mitochondrial cytochrome-C protein in the blood, during a decrease in the average density of mitochondria and the density of cristae, as well as a decrease in the expression of mitochondrial Mn-SOD, which may also provide insight into the specifics of oxidative stress activation after PH [42]. Also, several works have established that intrauterine hypoxia changes the expression profile of 48 genes associated with metabolic and oxidative stress, such as the subunit of glutathione-S-transferase and cytochrome-C-oxidase [43,44]. 

The decrease that we have detected in eNOS expression during a significant increase in the level of nitrotyrosine in both 1- and 2-month-old rats after intrauterine hypoxia has an explanation. The increased production of ROS in prenatal hypoxia leads to decreased NO bioavailability and suppression of eNOS expression [45,46]. An excess of NADPH during prenatal hypoxia is the cause of the formation of ROS, which can react with NO to form a stable peroxynitrite anion, reducing the bioavailability of NO [47]. 

**Figure 1:**
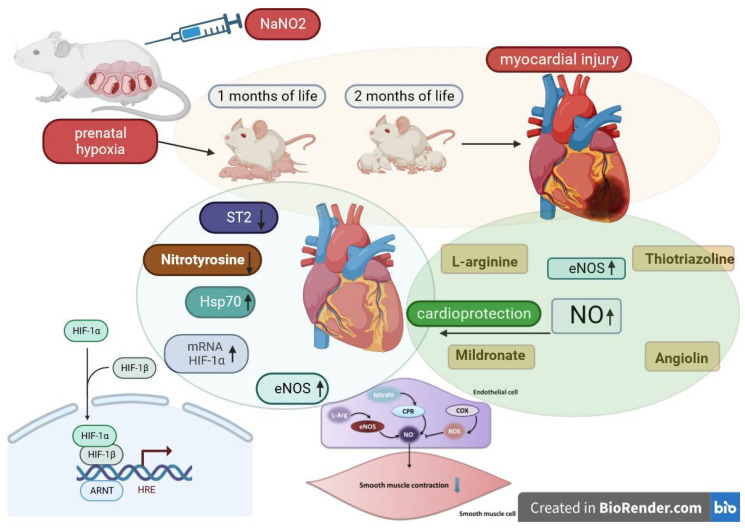
Cardiovascular system markers in blood and heart of rats following prenatal hypoxia and subsequent pharmacological modulation of the NO system.

Prolonged prenatal hypoxia leads to a decrease in HIF-1 mRNA expression in cells of other rat organs [48,49], and in our opinion, this may indicate a depletion of compensatory-adaptive reactions after intrauterine hypoxia. Hypoxia-induced factors (HIF) play the role of transcription factors and regulate the expression of genes encoding the synthesis of proteins involved in the physiological response to hypoxia/ischemia [50]. HIFs exhibit cytoprotective properties under hypoxia, stimulate reparative processes, and increase the concentration of free radical traps (haem-hydroxylase-1, haem-oxygenase-1, VEGF, angiopoietin) [51]. HIF-1 in conditions of hypoxia affects energy metabolism, regulating compensatory shunts of ATP synthesis, increases glutathione synthesis, and increases cell resistance to oxidative stress. HSP70 is known to prologue the “life time” of HIF-1. We found that suppression of HIF-1 mRNA expression after intrauterine hypoxia occurs against the background of HSP70 deficiency. A sufficient number of studies have shown multidirectional changes in the concentration of HIF-1, its forms at different types of hypoxia, its duration, and in different organs [52]. Under conditions of nitrosative stress and an increase in the level of cytotoxic products of NO and ATP deficiency in tissues, there is a decrease in HIF, associated with activation of ubiquitin-independent pathway of degradation of oxidatively modified HIF-1α and suppression of its synthesis at the stage of ATP deficiency. The regulatory role of NO in the regulation of HIF-1a mRNA expression is known [53,54,55]. Thus, one therapeutic strategy to reduce the cardiac dysfunction that develops after prenatal hypoxia may be to normalize the NO system and reduce oxidative stress (Figure 1). 

The therapeutic effect of the investigated pharmacological agents after PH can be explained as follows. The revealed primary cardioprotective effect of Angiolin, when administered after intrauterine hypoxia, with the preservation of the effect even after a monthly withdrawal, is explained by its following properties. Angiolin, under conditions of acute cerebral ischemia, exhibits pronounced endothelioprotective properties; it preserves the density of endotheliocytes, increases the concentration in the nuclei of RNA, increases the density of proliferating endotheliocytes (BrdU-test), increases the efficiency of utilization of endogenous L-arginine, and increases the expression of vascular endothelial factor (VEGF), as well as eNOS, and the presence of divalent sulfur in its structure determines the property of the NO scavenger [56]. 

In our work, it was found that Angiolin improved the ultrastructure of hippocampal CA1-zone neurons under conditions of chronic cerebral ischemia (reduced the destruction of cristae, uneven electron density of the matrix, increased the density of mitochondria), and also reduced the concentration of intramitochondrial iNOS and increased the concentration of cytological and intramitochondrial HSP70. It is known that 70 kDa heat shock proteins act as endogenous cytoprotectors during ischemia, hypoxia, exposure to toxins, and a sharp increase in temperature [57]. To date, it is known that the mechanisms of the protective action of HSP70 are realized due to the restoration of the correct tertiary structure of damaged proteins, as well as in the formation and dissociation of protein complexes. Our studies revealed the positive role of HSP70 aimed at reducing the formation of mitochondrial dysfunction in neurons of the sensorimotor cortex and hippocampus in cerebral ischemia [56].

In vitro experiments in a suspension of neurons in rat pups found that the introduction of HSP70 reduces the degree of damage to key enzymes of energy metabolism and enzymes of antioxidant protection of neuron enzymes. Also, it was found that HSP70 takes part in the regulation of the functioning of compensatory energy shunts in acute ischemia. We found that HSP70 “prolongs” the action of HIF-1a, and also independently maintains the expression of NAD-MDH-mx, thereby maintaining the activity of the compensatory mechanism of ATP production—the malate-aspartate shuttle mechanism—for a long time [58]. Our works have shown that Angiolin can activate the malate-aspartate shuttle mechanism in the myocardium during ischemia [56]. 

Thiotriazoline exhibits the properties of a scavenger of cytotoxic forms of NO and has a protective effect on NO transport, due to a positive effect on the thiol-disulfide balance and an increase in the level of reduced thiols and glutathione. In addition, we suggest that Thiotriazoline itself can be a NO carrier, forming stable S-nitrosyl complexes with it [59]. Thiotriazoline exhibits a cardioprotective effect, positively affecting energy metabolism in ischemic myocardium, increases ATP during ischemia and hypoxia due to the normalization of the Krebs cycle, increases the utilization of glucose and free fatty acids, and activates the conversion of lactate to pyruvate [60]. Thiotriazoline is also known to exhibit a cardioprotective effect and to increase the endurance of animals under working hypoxia by increasing HIF-1 and preserving mitochondrial ultrastructure. Due to the antioxidant action, Thiotriazoline maintains the threshold sensitivity of receptors, maintains membrane fluidity, and protects phospholipids from oxidation [61,62].

L-Arginine is a substrate for the formation of NO in vascular endothelial cells, a peripheral vascular dilatation factor. Formed from arginine NO, it reduces the total peripheral vascular resistance and blood pressure and reduces oxygen starvation, especially in the tissues of the heart [63]. The role of NO in the mechanisms of endotheliocyte proliferation and regulation of expression of vascular endothelial growth factor (VEGF), placental growth factor (PGF), angiopoietins (ANG-1, ANG-2), and receptor proteins (SFLT-1, STIE-2) is known. It has been shown that VEGF plays an important role in physiological pregnancy by regulating placental angiogenesis, reducing the incidence of placental insufficiency [64]. Physiological NO concentrations are known to regulate the expression of pro-angiogenic VEGF-A and PGF in vitro in human trophoblasts. Administration of iNOS inhibitors and a decrease in NO levels in pregnant mice resulted in an increase in blood pressure. NO at physiological concentrations can reduce the expression of pro-inflammatory cytokines and endothelial adhesion receptors and is capable of urgent expression of HIF-1a.

The properties of Mildronate have been studied for a long time. It is known that milronate through organic carnitine cation transporter 2 can reduce the level of L-carnitine and inhibit the transfer of fatty acids through mitochondrial membranes during acute ischemia or hypoxia. Mildronate prevents the accumulation of toxic metabolic intermediates acylcarnitine and acyl-CoA, which damage cell membranes and block the delivery of ATP from mitochondria to cell organelles [65]. Treatment with Mildronate is accompanied by a compensatory increase in the expression in the myocardium of a number of genes encoding lipid metabolism enzymes—lipoprotein lipase, fatty acid translocase, carnitine palmitoyltransferase I, and triacylglycerol synthesis enzymes. Mildronate is able to improve myocardial contractility, hexokinase activity, and the ratio of ATP/ADP/AMP by activating AMP-activated protein kinase, which restores ATP levels [66]. 

Mildronate can increase NO production in the ischemic myocardium and brain by modifying γ-butyrobetaine ester pools. Mildronate administration inhibits the hydroxylation of γ-butyrobetaine and increases the intracellular pool of γ-butyrobetaine, the esterification of which exhibits cholinomimetic properties. Esters of γ-butyrobetaine through acetylcholine receptors on endothelial cells can activate eNOS [67]. However, in a number of studies, the effect of Mildronate on NO production was not confirmed [7].

Thus, the results obtained established the primary cardioprotective effect of modulators of the NO system with different mechanisms of action—Thiotriazoline, Mildronate, L-arginine, and especially Angiolin—after prenatal hypoxia. The results obtained substantiate the prospects for further research.

## Figures and Tables

**Table 1:** Blood levels of cardiovascular markers and HIF-1a mRNA expression in the heart of animals after prenatal hypoxia and drug administration (1 month of life).

Experimental Groups *n* = 10	3-Nitrotyrosine nM/mL	ST_2_ ng/mL	eNOS, pg/mL	HSP_70_, ng/mL	mRNA HIF-1, o.u.
Intact	4.5 ± 0.82	16.7 ± 0.08	37.41 ± 0.88	14.42 ± 0.21	1.00 ± 0.0016
PH (control)	23.7 ± 1.23 ^1^	105.0 ± 3.94 ^1^	14.6 ± 0.26 ^1^	2.57 ± 0.12 ^1^	0.211 ± 0.0001 ^1^
PH + Angiolin	10.3 ± 0.87 ^1,^*	24.33 ± 2.35 ^1,^*	54.73 ± 1.02 ^1,^*	5.37 ± 0.17 ^1,^*	4.87 ± 0.005 ^1,^*
PH + Thiotriazoline	14.1 ± 1.18 ^1,^*	33.4 ± 0.56 ^1,^*	25.63 ± 1.31 ^1,^*	2.27 ± 0.12 ^1^	1.89 ± 0.001 ^1,^*
PH + L-arginine	18.4 ± 1.12 ^1,^*	28.33 ± 1.08 ^1,^*	32.4 ± 1.08 *	5.76 ± 0.19 ^1,^*	1.88 ± 0.001 ^1,^*
PH + Mildronate	20.2 ± 1.85 ^1^	32.17 ± 0.87 ^1,^*	28.4 ± 0.76 ^1,^*	3.73 ± 0.15 ^1^	0.37 ± 0.001 ^1^

*—*p* < 0.05 in relation to the indicators of the control group; 1—*p* < 0.05 in relation to the indicators of the intact group.

**Table 2:** Blood levels of cardiovascular markers and HIF-1a mRNA expression in the heart of animals after prenatal hypoxia and drug administration (2 months of life).

Experimental Groups *n* = 10	3-Nitrotyrosine nM/mL	ST_2_ ng/mL	eNOS, pg/mL	HSP_70_, ng/mL	mRNA HIF-1, o.u.
Intact	4.8 ± 0.77	14.7 ± 0.50	54.03 ± 0.47	14.9 ± 0.37 ^1^	1.00 ± 0.0003
PH (control)	18.2 ± 1.65 ^1^	53.33 ± 0.62 ^1^	26.43 ± 0.90 ^1^	4.33 ± 0.11	0.389 ± 0.0005 ^1^
PH + Angiolin	6.23 ± 0.76 *	12.1 ± 0.54 *	65.9 ± 0.65 ^1,^*	8.07 ± 0.15 ^1,^*	3.19 ± 0.001 ^1,^*
PH + Thiotriazoline	9.47 ± 1.14 ^1,^*	18.07 ± 0.88 ^1,^*	36.5 ± 1.04 ^1,^*	6.63 ± 0.13 ^1,^*	2.41 ± 0.0002 ^1,^*
PH + L-arginine	15.7 ± 1.35 ^1,^*	23.8 ± 1.00 ^1,^*	43.7 ± 1.30 ^1,^*	4.5 ± 0.19 ^1^	1.08 ± 0.00 ^1,^*
PH + Mildronate	16.4 ± 1.42 ^1^	26.33 ± 1.29 ^1,^*	26.2 ± 1.27 ^1^	6.33 ± 0.13 ^1,^*	1.88 ± 0.0005 ^1,^*

*—*p* < 0.05 in relation to the indicators of the control group; 1—*p* < 0.05 in relation to the indicators of the intact group.

## Data Availability

All the data generated during this research are included in the manuscript.
